# Detection, Distribution and Characterization of Novel Superoxide Dismutases from *Yersinia enterocolitica* Biovar 1A

**DOI:** 10.1371/journal.pone.0063919

**Published:** 2013-05-21

**Authors:** Mahesh Shanker Dhar, Vatika Gupta, Jugsharan Singh Virdi

**Affiliations:** Microbial Pathogenicity Laboratory, Department of Microbiology, University of Delhi South Campus, New Delhi, India; University of Helsinki, Finland

## Abstract

**Background:**

Superoxide dismutases (SODs) cause dismutation of superoxide radicals to hydrogen peroxide and oxygen. Besides protecting the cells against oxidative damage by endogenously generated oxygen radicals, SODs play an important role in intraphagocytic survival of pathogenic bacteria. The complete genome sequences of *Yersinia enterocolitica* strains show presence of three different *sod* genes. However, not much is known about the types of SODs present in *Y. enterocolitica*, their characteristics and role in virulence and intraphagocytic survival of this organism.

**Methodology/Principal Findings:**

This study reports detection and distribution of the three superoxide dismutase (*sodA, sodB* and *sodC*) genes in 59 strains of *Y. enterocolitica* and related species. The majority (94%) of the strains carried all three genes and constitutive expression of *sodA* and *sodB* was detected in 88% of the strains. Expression of *sodC* was not observed in any of the strains. The *sodA, sodB* and *sodC* genes of *Y. enterocolitica* were cloned in pET28a (+) vector. Recombinant SodA (82 kDa) and SodB (21 kDa) were expressed as homotetramer and monomer respectively, and showed activity over a broad range of pH (3.0–8.0) and temperature (4–70°C). SodA and SodB showed optimal activity at 4°C under acidic pH of 6.0 and 4.0 respectively. The secondary structures of recombinant SodA and SodB were studied using circular dichroism. Production of *Ye*SodC was not observed even after cloning and expression in *E. coli* BL21(DE3) cells. A SodA^−^ SodB^−^ *Escherichia coli* strain which was unable to grow in medium supplemented with paraquat showed normal growth after complementation with *Y. enterocolitica* SodA or SodB.

**Conclusions/Significance:**

This is the first report on the distribution and characterization of superoxide dismutases from *Y. enterocolitica.* The low pH optima of both SodA and SodB encoded by *Y. enterocolitica* seem to implicate their role in acidic environments such as the intraphagocytic vesicles.

## Introduction

*Y. enterocolitica* is an important food- and water-borne enteropathogen. It is associated with a variety of gastrointestinal problems and clinical manifestations that include acute gastroenteritis, terminal ileitis, and mesenteric lymphadenitis [1]. Virulence of *Y. enterocolitica* is attributed to the presence of a 70 kb pYV (*p*lasmid for *Yersinia v*irulence) plasmid and many chromosomally-encoded virulence factors [2]. It is an extracellular pathogen that has the ability to survive inside macrophages [3]. *Y. enterocolitica* can survive at low temperatures where its growth is governed by polynucleotide phosphorylase (PNPase) encoded by *pnp* gene [4]. Ability of *Y. enterocolitica* to grow at low temperatures and isolation from vacuum-packed frozen foods makes it an important pathogen associated with food-borne infections and poses a significant risk to the processed-food industry [5], [6]. Recently, Champion *et al*. [7] have reported that *Y. pseudotuberculosis sodC* mutant showed increased susceptibility to superoxide and reduced virulence in murine infection model. Similarly, intraphagocytic survival of *Y. enterocolitica* suggests resistance to reactive oxygen species (ROS) produced by macrophages although the mechanism has not been well defined. Roggenkamp *et al.* [8] have previously reported that SodA played an important role in the survival of *Y. enterocolitica* 1B/O:8 in the spleen and liver of mice and its absence led to an increased susceptibility of the organism to killing by neutrophils. However, no further studies have been reported on the nature and the distribution of superoxide dismutases from *Y. enterocolitica*.

Superoxide dismutases (EC 1.15.1.1) are metalloenzymes that detoxify oxygen radicals through the dismutation of superoxide to oxygen and hydrogen peroxide which is further reduced to water and oxygen by catalase or peroxidase [9]. These enzymes are ubiquitously present in both prokaryotes and eukaryotes where they function to protect the cells from endogenously generated superoxide anions during aerobiosis. Reactive oxygen species (ROS) such as superoxide anions (O^⋅^_2_ **)**, hydroxyl radicals (OH^⋅^) and hydrogen peroxide (H_2_O_2_) are known to damage nucleic acids, proteins and lipids [10], [11]. In addition to detoxification of endogenously produced superoxides, bacterial SODs play a significant role in the pathogenicity of animal and plant pathogens [12], [13], [14], [15]. Secretion of SODs by strains of *Brucella*, *Haemophilus*, *Legionella*, *Mycobacterium*, and *Nocardia*, further seems to suggest their role in neutralizing exogenous oxygen radicals produced by the host immune system [16]. On the basis of the metal co-factor, SODs are classified into four types: manganese co-factored (Mn-SOD or SodA), iron co-factored (Fe-SOD or SodB), copper-zinc co-factored (Cu/Zn-SOD or SodC) and nickel co-factored (Ni-SOD or SodN) [9], [17]. Prokaryotic SodA and SodB are located in the cytoplasm of the bacterial cell whereas SodC is periplasmic [9]. Cells that do not produce superoxide dismutase, in general, show reduced cellular health and increased damage due to oxidative stress. An increase in the frequency of the mutations has been observed in *E. coli* lacking SodA and SodB [18], and *Saccharomyces cerevisiae* lacking Mn-SOD were found to be highly sensitive to oxygen [19], [20].

In *Y. enterocolitica* the role of SodB and SodC has not been studied as yet. Moreover, SODs of *Y. enterocolitica* have not been characterized, till date. In this study, the distribution of *sod* genes in different strains of *Y. enterocolitica* biovar 1A was assessed. Furthermore, the SODs of *Y. enterocolitica* biovar 1A were cloned and expressed in *E. coli* BL21 (*DE3*) and the purified recombinant superoxide dismutases (*Ye*SODs) were extensively characterized biochemically and biophysically. This is the first report on the distribution and detailed characterization of novel SODs from strains of *Y. enterocolitica* biovar 1A.

## Materials and Methods

### 2.1. Bacterial Strains and Vectors

A total of 54 strains of *Y. enterocolitica* were used in this study. Three strains of *Yersinia intermedia* and two of *Yersinia frederiksenii* were also included in the study. The details of these strains are given in supplementary data (Table S1 in File S1). The strains were grown overnight in tryptone soya broth (TSB) or tryptone soya agar (TSA) plates (HiMedia, Mumbai, India) at 28°C. *E. coli* strains were grown overnight in Luria Bertani (LB) broth at 37°C with shaking. Details of strains and vectors used in cloning and expression of *sod* genes are given in Table 1.

**Table 1:** Bacterial strains and plasmids used for cloning and expression in this study.

Strains/Plasmids	Relevant characteristics	Reference/Source
***Y. enterocolitica***		
IP27366	Biotype 1A; serotype O:6,30–6,31, clinical isolate	[68]
***E. coli***		
DH5α	*F2 D(lac-argF)U169 recA1 endA1 hsdR (rK 2 mK 1) supE44 gyrA1 relA1 deoR thi-1 (F80dlacZDM15)*	Invitrogen
BL21(*DE3*)	*E. coli* B F^−^ dcm ompT hsdS(r^−^_B_ m^−^_B_ ) gal λ(DE3)	Stratagene
AB1157	*F- thr-1; leuB6; proA2; his-4; thi-1; argE2; lacY1; galK2; rpsL; supE44; ara-14; xyl-15; mtl-1; tsx-33*	Joan S. Valentine^a^
PN134	AB1157 with (*sodA::Mu d PR13)25 (sodB−kan)*	Joan S. Valentine^a^
**Plasmids**		
pGEM-T easy	T-A Cloning vector; *Amp* ^r^	Promega
pGEMsodA	pGEM cloning vector with 624 bp fragment of *sodA* amplified from *Y. enterocolitica*	This study
pGEMsodB	pGEM cloning vector with 579 bp fragment of *sodB* amplified from *Y. enterocolitica*	This study
pGEMsodC	pGEM cloning vector with 525 bp fragment of *sodC* amplified from *Y. enterocolitica*	This study
pGEMsodCTrunc	pGEM cloning vector with 471 bp truncated fragment of *sodC* amplified from *Y. enterocolitica*	This study
pET28a^+^	pET vectors are derived from pBR322 with opposite orientation of the cloning/expression region; Kan^r^	Novagen
pET28sodA	pET28 vector containing *YesodA*	This study
pET28sodB	pET28 vector containing *YesodB*	This study
pET28sodC	pET28 vector containing *YesodC*	This study
pET28sodCTrunc	pET28 vector containing truncated copy of *YesodC*	This study
pGFPuv	GFP gene cloned between the two MCSs of the pUC19 derivative pPD16.43, *Amp* ^r^	Clontech
pGFPsodA	pGFP vector containing *YesodA*	This study
pGFPsodB	pGFP vector containing *YesodB*	This study

a Prof. Joan S. Valentine, University of California Los Angeles.

### 2.2. PCR Detection of *sodA, sodB* and *sodC* Genes in *Yersinia* spp

Genomic DNA from the strains was isolated using Pure Link Genomic DNA Minikit (Invitrogen, USA), as per manufacturer’s instructions and used as the template for amplification of *sodA*, *sodB* and *sodC* by PCR. The details of the primers and the respective PCR conditions used are given in Table 2. The primers were designed using the complete genome sequence of *Y. enterocolitica* strain 8081 (accession no. AM286415).

**Table 2:** Details of primers and the PCR conditions.

Gene	Primers	Sequence (5'-3')	Amplicon(bp)	Annealing temperature	Restrictionsites	Position of primers on the *Y.e.* strain 8081 complete genome
*sodA*	SodA 624F	TTT**GGATCC**ATGAGTTACTCACTGCCATCCCT	624	58°C –40s	*Bam*HI and *Sac*I	4530100–4530122
	SodA 624R	TTT**GAGCTC**CTTAGCTTGAGCGAAGCGC				4530720–4530702
*sodB*	SodB 579F	TTT**GAGCTC**ATGTCTTTTGAATTACCGGCGTT	579	59°C –40s	*Sac*I and *Not*I	2356988–2357007
	SodB 579R	TTT**GCGGCCGCCC**GGCTAAGTTTTTCTCAACGAA				2357563–2357543
*sodC*	SodC 525F	AAA**GCGGCCGC**ATGAAACTGAAATACTTAGTGTTACCC	525	50°C –40s	*Not*I and *Xho*I	877988–878014
	SodC 525R	AAA**CTCGAG**TTACTCAATCACACCACAAGC				878514–878492
*sodC*	SodC Trun F	AAA**GCGGCCGC**ATGGCTGCGGATATTACTGTGAC	476	50°C –40s	*Not*I and *Xho*I	NA
	SodC 524R	AAA**CTCGAG**TTACTCAATCACACCACAAGC				878514–878492

Nucleotide bases in bold represent restriction sites. NA: the primer was designed using the sequence of *sodC* (accession no. JX204785) from strain IP27366 as the template.

### 2.3. Whole Cell Protein Extraction and Zymogram Analysis

Overnight grown cultures of *Yersinia* spp. were harvested, washed and resuspended in lysis buffer (50 mM Tris-HCl, 10 mM MgCl_2_, 1 mM EDTA) containing 4 mM/mL PMSF (phenyl methane sulfonyl chloride). The cells were lysed by sonication on ice with 5 cycles of 1 min pulse (3 sec on/off) to obtain the cytoplasmic proteins. The periplasmic proteins were obtained by suspending the cell pellet in 20 mM TE buffer (pH 8.0) containing 25% (w/v) sucrose and 1 mM EDTA. The cell suspension was incubated at 30°C with mild shaking for 15 min. The cells were collected by centrifugation at 4°C followed by osmotic treatment for 10 min by suspending the cell pellet into 5 mM chilled MgSO_4_ solution to release the periplasmic fraction of the cells which was further collected as supernatant on centrifugation. The total protein concentration of each bacterial lysate was estimated using Bradford method [21] with bovine serum albumin (BSA) (0–100 mM) as standard.

The presence of active SODs was confirmed by zymogram analysis on native PAGE using the methodology described previously [22], with slight modifications. Crude protein extracts (cytoplasmic or periplasmic) were electrophoresed on non-denaturing 15% polyacrylamide (1∶29, bisacrylamide-acrylamide) gel using a mini-Protean III apparatus (Bio-Rad, USA). The gels were soaked in 2.4 mM nitrobluetetrazolium (Sigma) for 25 min in dark and later immersed in 0.03 mM riboflavin solution containing 210 µl tetramethylethylenediamine (TEMED) for 25 min followed by illumination of gels under a 15 W luminescent light source for 20 min.

### 2.4. Differentiation of SodA, SodB and SodC

Enzyme inhibitors were used to differentiate different SODs expressed by the *Y. enterocolitica* strains. Following separation of the total cell protein on non-denaturing PAGE, the gels were treated with H_2_O_2_ [23] and sodium azide [24] to inactivate Fe-SOD, whereas, diethyldithiocarbamate (DDTC) was used to inactivate the Cu/Zn-SOD [25]. After 30 min of incubation with different inhibitors, the gels were washed thoroughly to remove the inhibitors and processed for zymogram development.

To detect the presence of Cu/Zn-SOD, ice cold 2∶3 v/v chloroform: ethanol solution was added to the crude protein as described previously [26]. The protein-chloroform:ethanol mixture was kept at room temperature for 30 s and centrifuged at 2,500 g for 10 min. The aqueous phase, devoid of Mn- and Fe-SOD, was carefully loaded on the native gel and processed for zymogram development.

### 2.5. RT-PCR for SOD mRNA Transcripts

RNA was extracted from overnight cultures of *Y. enterocolitica, Y.* *intermedia* and *Y. frederiksenii* grown in the presence or absence of paraquat (Sigma, USA), using RNeasy Mini Kit (Qiagen, USA) according to the manufacturer’s instructions. Paraquat (methyl viologen) (0.1 mM) was added to the culture media to induce oxidative stress conditions for the bacteria. 1 µg total RNA was used for single-strand cDNA synthesis using iScript cDNA synthesis kit (Bio-Rad, USA), according to manufacturer’s instructions. The cDNA was further used to amplify *sodA*, *sodB* and *sodC* using specific primers (Table 2).

### 2.6. Expression and Purification of Recombinant Superoxide Dismutases

*sodA, sodB* and *sodC* genes were amplified from *Y. enterocolitica* strain IP27366, cloned in pGEM-T easy vector and propagated in *E. coli* DH5α cells. Cloning of *sod* genes was confirmed using colony PCR followed by sequencing using Sanger dideoxy sequencing method at the central instrumentation facility, University of Delhi South Campus using ABI 3700 genetic analyzer. Further, the respective *sod* genes were subcloned into pET28a (+) vector at specific restriction sites (*sodA*- *Bam*HI-*Sac*I; *sodB*- *Sac*I-*Not*I and *sodC*- *Not*I-*Xho*I) using instant ligation mix (Clontech, Takara). Thereafter, the recombinant plasmids (pET28a-*sodA/sodB/sodC*) were transformed separately into *E. coli* BL21 (*DE3*) and confirmed by sequencing. The positive clones were grown overnight. Erlenmeyer flask (250 mL) containing 50 mL of LB broth supplemented with kanamycin (50 µg/mL) were inoculated with respective cultures grown overnight [1% (v/v)] and incubated till the optical density of 0.5 at 600 nm was achieved. The expression of recombinant SODs was induced by addition of 1 mM isopropyl-β-D-1-thiogalactopyranoside (IPTG) to the cultures which were incubated further. Cells were harvested periodically to study the expression of the recombinant proteins.

The recombinant *Y. enterocolitica* SODs (*Ye*SodA and *Ye*SodB) were purified by affinity chromatography using Ni^2+^-NTA agarose resins (Novagen, USA) according to manufacturer’s instructions. Purified recombinant proteins were eluted in 5 mL of 250 mM imidazole and analyzed on 15% SDS-PAGE to check the purity and titre of the recombinant SODs. Further, the molecular weights of the purified proteins were determined by SDS-PAGE and fast protein liquid chromatography (FPLC, AKTAPrime plus). FPLC was performed using a Sephacryl S-200 column (16/60, AKTA) pre-equilibrated with 100 mM potassium phosphate buffer (pH 7.5) [27].

### 2.7. Biochemical Characterization

Isoelectric focusing (IEF) of the purified enzymes (*Ye*SodA and *Ye*SodB) was carried out in 6% polyacrylamide gel containing 2% ampholyte (pH 5–7) (Biolyte, Ampholyte, Bio-Rad). 3–5 µL of purified protein containing *ca*. 5 µg of protein was loaded on the gel and focused using a Mini IEF cell (Bio-Rad, USA) at 4°C according to the manufacturer’s instructions. The gels were stained with coomassie brilliant blue stain to visualize the SOD bands against the pI ladder.

Thermostability and pH stability were measured by incubating purified *Ye*SODs at different temperatures (4–70°C) and pH (2–10) for varying time intervals (5 min – 24 h) followed by determining the enzyme activity using superoxide dismutase kit (Cayman Chemicals, USA) according to manufacturer’s instructions.

### 2.8. Biophysical Characterization using Circular Dichroism (CD)

The secondary structures of *Ye*SodA and *Ye*SodB were analysed from the CD spectra in the ‘far-UV’ spectral region (190–240 nm) using a JASCO J-815 spectropolarimeter equipped with a peltier thermostatic cell holder (PTC-348 WI, JASCO, Japan). The far-UV CD spectrum was recorded as described previously [28]. The proteins in different buffers of pH (3.0–8.0) were scanned at different temperatures. Results were expressed as mean residue ellipticity by calculating mean residue weight per amino acid residue. The K2D2 software [29] was used for analyzing the data.

### 2.9. Growth of *E. coli* SodA^−^ SodB^−^ Strain Complemented with *Ye*SodA and *Ye*SodB under Oxidative Stress

The effect of paraquat was observed on the growth of SodA^−^ SodB^−^ *E. coli* strain PN134, complemented with *Ye*SodA or *Ye*SodB. Full length *sod*A and *sod*B genes from *Y. enterocolitica* strain IP27366 were cloned into pGFPuv vector (Clontech). The individual recombinant vectors (pGFP*sodA* or pGFP*sodB*) were transformed into *E. coli* PN134. The *E. coli* PN134, expressing *Ye*SodA or *Ye*SodB, was propagated aerobically at 37°C overnight in LB broth (25 ml) supplemented with 0.2% sucrose. The overnight cultures were subcultured into fresh LB broth (250 mL) and kept at 37°C with agitation at 200 rpm in an orbital shaker (New Brunswick Scientific, USA) to A_600_ of 0.2. The culture was divided equally into five 250 mL flasks, each containing a different concentration of paraquat and incubated at 37°C with agitation. The bacterial growth was monitored every 2 h by measuring A_600_ with a spectrophotometer. A wild type strain (*E. coli* AB1157) and *E. coli* PN134 (SodA^−^ SodB^−^) were also included as controls.

### 2.10. Proposed 3D Structures of *Y. enterocolitica* SODs

Crystal structures of SODs (PDB ID: 1VEW and 1ISA) from *E. coli* were used to propose the secondary and tertiary structure of *Ye*SODs [30], [31]. Preliminary studies on the secondary structures were carried out using online software ESpript 2.2. The deduced amino acid sequences of *Ye*Sods were submitted to online server ESyPred3D Web Server 1.0 [32] to obtain a PDB file. PyMol software was used to view the PDB files for constructing the 3D structure.

### 2.11. Nucleotide Sequence Accession Numbers

The *Y. enterocolitica sodA, sodB* and *sodC* sequences were submitted to GenBank database and assigned accession nos. JX204782, JX204783 and JX204785 respectively.

## Results

### 3.1 Distribution and Expression of *sod* Genes

PCR amplifications revealed that *sodA* gene was present in all strains, *sodB* was absent only in one strain (*Y. enterocolitica* biovar 1A strain IP27430) whereas *sodC* was absent in two *Y. intermedia* strains (IP27388 and IP27389) (Table S2 in File S1).

**Figure 1:**
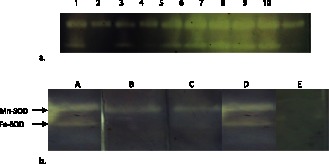
Zymogram analysis of superoxide dismutases: (**a**) Representative zymogram showing two acromatic bands of crude bacterial lysate from *Yersinia* spp. (**b**) Effect of different inhibitors on SOD isoforms of *Y. enterocolitica*. Lane A: Cell lysate without inhibitor; B: with 5 mM H_2_O_2_; C: with 5 mM NaN_3_; D: with DDTC; E: with chloroform:ethanol.

Zymogram analysis showed two achromatic zones in each lane instead of the expected three clear zones (Figure 1a). Treatment of *Y. enterocolitica* crude lysate with specific SOD inhibitors (Figure 1b) revealed expression of SodA and SodB in 100% and 88% of the strains respectively, whereas SodC was not expressed by any of the strains (Table S2). Additionally, growth even under conditions of oxidative stress and enrichment of growth medium by incorporation of Cu/Zn (0.1–2 mM) did not induce the expression of SodC.

RT-PCR not only generated amplicons of *sodA* (624 bp) and *sodB* (579 bp) as expected, but also amplified a 525 bp (*sodC*) amplicon when primers specific for *sodC* were used. The *sodC* was amplified from cDNA from strains grown with or without paraquat in the culture medium.

### 3.2. PCR Amplification and Sequence Analysis

**Figure 2:**
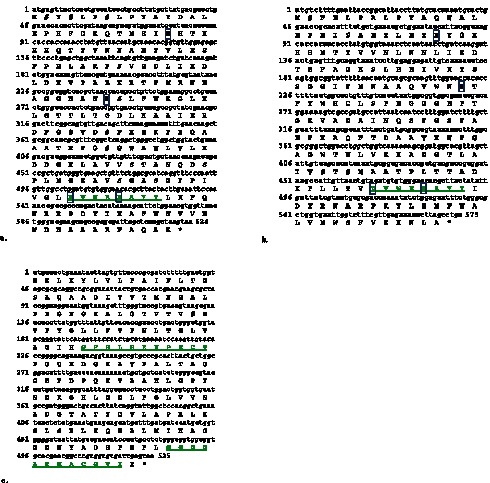
Nucleotide and deduced amino acid sequences of the superoxide dismutase genes from *Y. enterocolitica* strain IP27366: (**a**) SodA showed one signature sequence (D V W E H A Y Y) and four metal binding ligands (H-27, H-82, D-169 and H-173). (**b**) SodB also showed one signature sequence (D V W E H A Y Y) and four metal binding ligands (H-27, H-74, D-157and H-161). The metal binding ligands are shown in boxes. (**c**) SodC showed signature sequence I (G F H L H E N P S C T) and II (G G G G A R M A C G V I).

Full-length *sod* genes of *Y. enterocolitica* strain IP27366 were amplified using specific primers and cloned into pET28a(+) vector. The sizes of the *Y. enterocolitica sodA, sodB* and *sodC* genes were 624 bp, 579 bp and 525 bp with an overall G+C content of 51%, 46% and 50% respectively. The deduced amino acid sequences revealed presence of the signature sequences of the respective SOD families (Figure 2). Phylogenetic analysis showed proximate relationships of *Ye*SODs and other bacterial species based on amino acid sequence of respective SOD enzymes (Figure S1).

### 3.3. Expression and Purification of Recombinant *Ye*SODs

**Figure 3:**
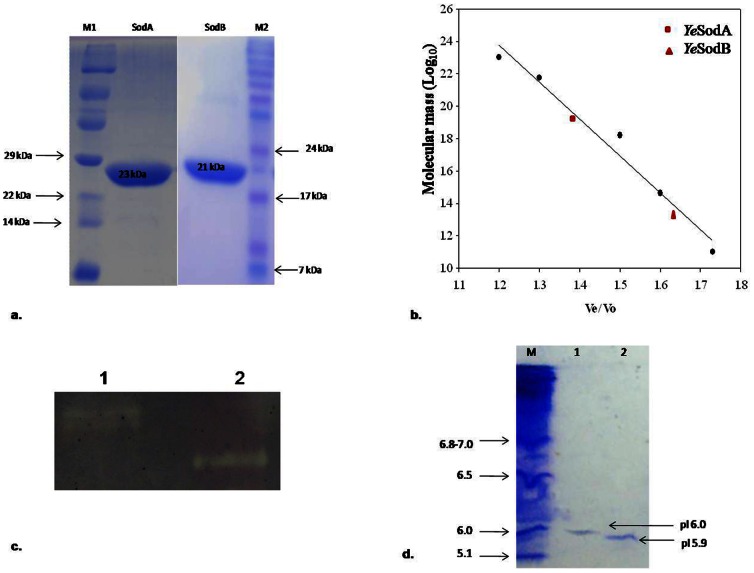
Molecular weight, activity and pI analysis of recombinant SODs: (**a**) SDS–PAGE of recombinant *Ye*SodA and *Ye*SodB expressed in pET 28a (+) (samples were resolved on 15% polyacrylamide gel and stained with Coomassie Brilliant Blue R-250). The purified SodA and SodB showed a single band each of 23 KDa and 21 kDa respectively. M1 and M2: Protein marker; Lane 1: SodA; Lane 2 SodB. (**b**) Molecular weight determination of *Ye*SodA (82 kDa) and *Ye*SodB (21 kDa) by Sephacryl S-200 molecular sieve chromatography. The molecular weight of marker proteins (SigmaAldrich) were as follows: β-Amylase (200 kDa), Alcohol dehydrogenase (150 kDa), BSA (66 kDa), Carbonic anhydrase (29 kDa) and Cytochrome C (12.4 kDa). (**c**) Zymogram analysis showing achromatic bands of *Ye*SodA and *Ye*SodB against a dark background. Lane 1: *Ye*SodA; Lane 2: *Ye*SodB. (**d**) Isoelectric point (pI) of purified recombinant *Ye*SodA and *Ye*SodB stained with coomassie brilliant blue. M: pI marker; Lane 1: *Ye*SodA; Lane 2: *Ye*SodB.

The expression of recombinant *Ye*SodA and *Ye*SodB was induced by addition of 1 mM IPTG in *E. coli* BL21 (*DE3*). These were purified using Ni^2+^-NTA resins and observed as single protein band each on 15% SDS-PAGE (Figure 3a). Molecular weights of *Ye*SodA and *Ye*SodB were observed to be 23 kDa and 21 kDa respectively on SDS-PAGE. Further, analysis of purified *Ye*SodA and *Ye*SodB by FPLC revealed molecular weight of 82 kDa and 21 kDa respectively (Figure 3b). This suggested that *Ye*SodA and *Ye*SodB were expressed as tetramer and monomer respectively. However, SodC was not expressed even after cloning in pET28a(+) vector.

Multiple sequence alignment of the deduced amino acid sequence of SodC showed highly unconserved N-terminal region. Therefore, an attempt was made to truncate *sodC* for 54 nucleotides downstream of the initiation codon and was amplified using specific primers (Table 2). The truncated gene was cloned in pET28a (+) vector and transformed into *E. coli* BL21 (*DE3*). However, even the truncated recombinant *sodC* could not be expressed after IPTG induction.

The zymogram analysis of the purified *Ye*SodA and *Ye*SodB revealed clear bands against the dark background of the polyacrylamide gel which further confirmed the expression of active recombinant *Ye*SodA and *Ye*SodB (Figure 3c).

### 3.4. Biochemical Characterization

The isoelectric points (pI) of the native *Ye*SodA and *Ye*SodB were observed to be approximately 6.0 and 5.9 (Figure 3d), respectively. These values were quite similar to the theoretical pI’s of 6.2 and 5.8 respectively, which calculated by PROTPARAM software (http://web.expasy.org/protparam).

**Figure 4:**
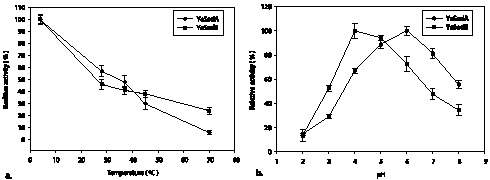
Effect of physical parameters on recombinant SOD activity: (**a**) Optimum temperature of *Ye*SodA and *Ye*SodB was 4°C (**b**) while optimum pH was 4.0 and 6.0 respectively. The results are expressed as percent change in the activity of the respective enzyme with the value at optimum temperature and pH taken as 100%.

The recombinant *Ye*SodA and *Ye*SodB were active over a broad range of pH (3.0–8.0) and temperature (4–70°C) with optimum of *Ye*SodA at pH 6.0 and 4°C and, that of *Ye*SodB at pH 4.0 and 4°C. The specific activities of purified *Ye*SodA and *Ye*SodB at optimum temperature and pH were 12,800 U/mg and 24,400 U/mg of protein respectively. The activities of these enzymes decreased steadily with increase in temperature whereas change in pH also had significant effect on the enzyme activity (Figure 4).

### 3.5. Proposed 3D Structure

**Figure 5:**
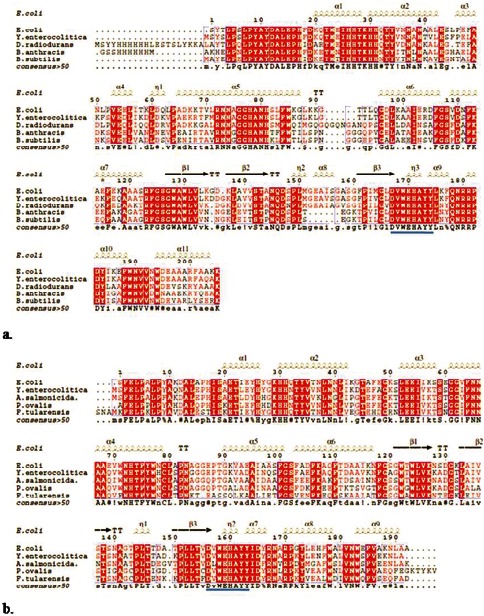
Sequence homology: Multiple sequence alignment (MSA) of (**a**) *Ye*SodA with *E. coli* (PDB id: 1VEW), *Deinococcus radiodurans* (PDB id: 2CDY), *B. anthracis* (PDB id: 1XUQ) and *B. subtilis* (PDB id: 2RCV); (**b**) *Ye*SodB with *E. coli* (PDB id: 2NYB*), Aliivibrio salmonicida* (PDB id: 2W7W), *Pseudomonas ovalis* (PDB id: 1DT0) and *Francisella tularensis* (PDB id: 3H1S) drawn using ESPript 2.2. Symbols α and β indicate alpha helices and beta sheets, respectively; η represents turns and TT denotes sharp turns in the structure.

ESpript 2.2 analysis showed α-helix dominant structures of *Ye*SodA and *Ye*SodB. Preliminary structure of *Ye*SodA comprised of 11 α-helix and 3 β-sheets, whereas, *Ye*SodB showed a total of 9 α-helix and 3 β-sheets along with random coils and sharp turns in both enzyme structures (Figure 5).

**Figure 6:**
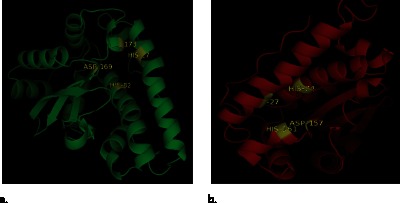
Proposed three dimensional structure: Predicted 3D structure of (**a**) SodA and (**b**) SodB showing metal binding ligands: His27, His82, Asp169 and His 173 in SodA and, His27, His74, Asp157 and His161 in *Ye*SodB.

The proposed three dimensional structures of *Ye*SodA and *Ye*SodB imitate similar profile of α-helix and β-sheets. Sequence analysis revealed metal binding ligands in *Ye*SodA as His27, His82, Asp169 and His173, and in *Ye*SodB as His27, His74, Asp157 and His161. Two of these amino acids (Asp169/His173 for SodA and Asp157/His161 for SodB) were present in the highly conserved signature sequences. 3D structure analysis showed clustering of all four metal binding ligands which formed a groove for binding the respective metal ion (Figure 6).

### 3.6. Biophysical Characterization

#### 3.6.1. Secondary structure

**Figure 7:**
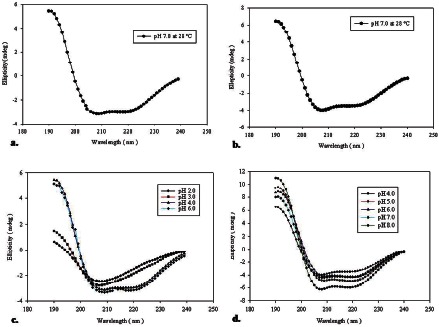
Secondary structure analysis using circular dichroism: Far-UV spectra of (**a**) *Ye*SodA and (**b**) *Ye*SodB at pH 7 and 28°C. Far-UV spectra of (**c**) *Ye*SodA and (**d**) *Ye*SodB at different pH.

The secondary structures of purified *Ye*SodA and *Ye*SodB were determined by circular dichroism spectroscopy (CD). The far-UV CD spectra indicating the secondary structures of *Ye*SodA and *Ye*SodB are presented in Figure 7. The secondary structure of SodA at 28°C and pH 7.0 showed 29% α-helices and 16% β-sheets whereas SodB consisted of 44% α-helices and 13% β-sheets (Figure 7a, b). The far-UV CD spectra of the purified proteins recapitulate the predictions from ESpript 2.2 and proposed 3D structure of the respective SODs. Although no significant changes were observed in the secondary structures of the SODs with increase in temperature (data not shown), change in pH had considerable impact on the α-helix and β-sheet content of the respective SODs (Figure 7c, d).

### 3.7. Significance of *Y. enterocolitica* SodA and SodB in Growth under Oxidative Stress

**Figure 8:**
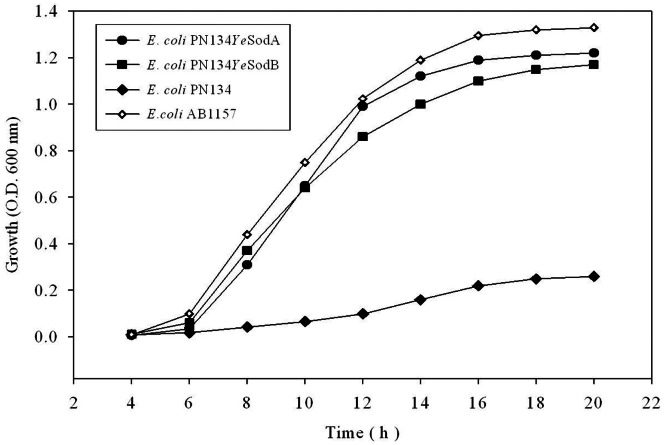
Analysis of Growth profile: Growth of *E. coli* PN134 (SodA^−^ SodB^−^), wild type *E. coli* AB1157 (SodA^+^ SodB^+^) and *E. coli* PN134 complemented with *Ye*SodA or *Ye*SodB in the presence of paraquat (0.1 mM).

The effect of paraquat on growth of *E. coli* strain PN134 expressing *Ye*SodA or *Ye*SodB was studied. Figure 8 shows effect of paraquat on the survival of *E. coli* PN134 expressing *Ye*SodA and *Ye*SodB compared to a wild type strain of *E. coli* (AB1157) and the SOD double mutant (*E. coli* PN134 SodA^−^ SodB^−^). The dose-dependent inhibition of growth indicated that all recombinant strains were inhibited by high concentration of paraquat (data not shown); however, at lower concentrations (0.1 mM) the strain expressing *Ye*SODs showed resistance to paraquat-induced oxidative stress. *E. coli* PN134 SodA^−^ SodB^−^ strain showed high sensitivity even to low concentrations of paraquat (0.1 mM) whereas the wild type strain displayed normal growth (Figure 8).

## Discussion

In this study, we report PCR detection, distribution and expression of different superoxide dismutases in strains of *Y.* *enterocolitica* and related species. PCR amplification revealed presence of three *sod* genes in majority (95%) of the strains used in this study. The assays to detect superoxide dismutase activities revealed that each strain expressed at least one functional SOD. Specific inhibitors were used to distinguish the types of the superoxide dismutases expressed by the strains. The Mn- and Fe-SODs of *Y. enterocolitica* were inhibited by chloroform:ethanol solution as reported previously [26], [33]. Inhibition of the band with lower molecular weight by sodium azide and hydrogen peroxide confirmed the presence of Fe-SOD [23], [24]. The failure of diethyldithiocarbamate (DDTC) to inhibit any of the superoxide dismutases suggested absence of the active SodC and confirmed that the high molecular weight achromatic band was Mn-SOD [25].

Presence of identical signature sequences and similar conserved metal binding domains seemed to suggest that *Y. enterocolitica* SodA and SodB originated from a common progenitor gene as reported earlier by Smith and Doolittle [34]. The deduced amino acid sequence of *Ye*SodA was the most conserved of the three SODs. Phylogenetic analysis of *Ye*SodA indicated high (>82%) similarity with SodA from other gram-negative enteropathogens and shared 86% and 47% similarity with *E. coli* SodA and human mitochondrial Mn-SOD, respectively. BLAST analysis of *Ye*SodA revealed 66% similarity with *Deinococcus radiodurans* [35], a highly radio resistant organism. However, the significance of this similarity is not clear and requires further investigation. *Ye*SodB showed similarity with Fe-SODs encoded by members of the family Enterobactericeae and shared *ca.* 82% similarity with *E. coli* SodB. Analysis of deduced amino acid sequence of *Ye*SodC indicated that it was the least conserved of the three *Ye*SODs and showed low similarity with strains of other *Yersinia* spp. and only 58% similarity with the *E. coli* SodC. The N-terminal region of SodC was highly unconserved. Although, *Ye*SodA and *Ye*SodB showed high similarities to superoxide dismutases from members of gamma-proteobacteria only, *Ye*SodC showed similarity to that of members of the alpha-proteobacteria such as *Brucella* spp.

Benov and Fridovich (1994) [36] reported that SodC activity in *E. coli sodA* ^−^ *sodB* ^−^ double mutant was *ca.* 2% of the total SOD activity of a wild type strain. To verify whether *Ye*SodC was expressed at such low concentrations in our studies, the complete *sodC* gene was cloned and transformed in *E. coli* BL21 (*DE3*) cells. However, expression of SodC was not detected even after induction with IPTG. The 5′ region that encodes the signal sequence was highly unconserved in *sodC* and might be the reason for non-expression of SodC. The presence of SodC in the periplasmic space, as seen in other organisms, reiterates the importance of the signal peptide in guiding the enzyme to its required destination. Therefore, highly variable N- terminal region of SodC was truncated and tried to express in *E. coli* BL21 (*DE3*) cells however, no expression was detected. Enrichment of growth medium with incorporation of Cu/Zn also failed to express SodC. In addition, growing *Y. enterocolitica* in the presence of different concentrations of paraquat did not lead to “oxidative stress-induced” expression of SodC as reported for *Brucella abortus* [37], *B. melitensis* [38] and *Caulobacter crescentus* [39]. Nevertheless, RT-PCR revealed transcription of SodC mRNA when *Y. enterocolitica* was grown under normal conditions. It has also been previously reported that bacterial SodC showed wider variations in their amino acid sequences, compared to eukaryotic SodC which share a single structural model that has been preserved strictly throughout the evolution [40]. It has also been observed that bacterial SodC showed high degree of insertions and deletions in the major loops of the β-barrel that may cause disparity in the conformation of the active-sites and their subunit assembly. Substitutions of conserved metal ligands may also lead to significant alterations in the enzyme activity [41], [42]. Therefore, in the light of the constitutive transcription of *sodC* mRNA in *Y.* *enterocolitica*, we may hypothesize that the highly unconserved N-terminal region of the *Ye*SodC may hinder proper folding of the protein leading to its degradation. Similarly, Bakshi *et al.* [43] reported that *Francisella tularensis sodC* does not encode a functional protein under any set of growth conditions, whereas, expression of SodB was observed throughout the growth phase. Interestingly, similar to the *Ye*SodC, BLASTP analysis of *F. tularensis* SodC showed highly unconserved N- terminal region that, however, contains excellent signal peptide characteristics. These observation, although not conclusive, point towards an important role of N- terminal signal sequence in the expression of SodC.

The active recombinant *Ye*SodA and *Ye*SodB proteins were obtained in solublized form from the cytoplasmic fraction of the cells which was consistent with a previous report [44]. The predicted molecular masses deduced from amino acid sequences of *Ye*SodA and *Ye*SodB were 23 and 21 kDa respectively. However, FPLC analysis revealed molecular masses of 82 kDa and 21 kDa of *Ye*SodA and *Ye*SodB respectively, which suggested that *Ye*SodA was expressed as tetramer whereas *Ye*SodB was expressed as a monomer although its homologs in most prokaryotes are expressed as dimers [45], [46], [47] or tetramers [48], [49]. Similarly, prokaryotic SodC, which is dimeric in most organisms, has been reported to be expressed as monomers in *E. coli* [50] and *Salmonella* [51].

The optimal temperature and pH of *Ye*SodA was observed to be 4°C and pH 6.0; for *Ye*SodB it was 4°C and pH 4.0. At optimal pH and temperature *Ye*SodA and *Ye*SodB showed specific activities of 12,800 U/mg and 24,400 U/mg, respectively. The low temperature optima of *Ye*SodA and *Ye*SodB were exceptional as most SodA and SodB which have been characterized in the past showed temperature optima of 25–70°C. Fe-SOD has been reported to show optimum activity at 4°C in only a few bacteria such as *Aliivibrio salmonicida* [52] and *Pseudoalteromonas haloplanktis* [53]. Similarly, a low pH optimum for bacterial SODs has been reported only rarely. Wang *et al.* [54] reported that *Rhodothermus* Fe-SOD showed optimum activity at pH 5.0 and 50°C, whereas Lumsden *et al.* [55] characterized a Mn-SOD from *Rhodopseudomonas* that retained 70% of its activity after 30 min at pH 3.0. The low pH optima of both *Ye*SODs may be of significance under acidic conditions such as those encountered inside the macrophages and eventually help in the dissemination of *Y. enterocolitica* in the host by sequestering the organism in these cells. At low temperatures, reduction in the cellular metabolic rate causes accumulation of electrons in the respiratory chain that leads to overproduction of reactive oxygen species [56]. *Y. enterocolitica* being a psychrotrophic food-borne pathogen, the low temperature optima of *Ye*SODs may help in neutralizing the excess oxygen radicals produced during its growth at lower temperatures in refrigerated foods [57]. Therefore, we may hypothesize that the SODs produced by *Y. enterocolitica* with high specific activities are helpful in dismutating endogenously produced reactive oxygen species at low temperatures. Both the *Ye*SODs were also endowed with significant overall thermal stability which is common for SODs belonging to this family.

The pIs of *Y. enterocolitica* SodA (6.0) and SodB (5.9) were almost similar to the theoretical pI of 6.2 and 5.8, respectively, and were in the range of pI values (3.8–6.0) for the known respective superoxide dismutases [58], [59].

The conservation of most of the amino acid residues of *E. coli* SODs involved at active site, substrate binding and catalysis in *Ye*SodA and *Ye*SodB as well, suggested similar structure and function. The metal binding residues- His27, His82, Asp169 and His173 in *Ye*SodA were also conserved as reported earlier for *Thermus thermophilus*, *Bacillus stearothermophilus* and *Bacillus subtilis* [46], [49], [60]. Similarly, in *Ye*SodB His27, His74, Asp157 and His161 were in accordance with conserved residues in *E. coli*, *Helicobacter pylori* and *Pseudomonas ovalis* [61], [62], [63].

Analysis of far-UV CD spectra of SodA and SodB revealed that the elements of secondary structures were more sensitive to pH than to temperature. At pH 7.0 and 28°C, SodA contained 29% α-helix and 16% β-sheets whereas SodB contained 44% α-helix and 13% β-sheets. The α-helix (29%) in SodA agreed with the 30% found in *E. coli* Mn-SOD than 40–50% seen in the crystal structures of the Mn-SODs from *Thermus thermophilus* and *Bacillus stearothermophilus* [45], [46], [49]. The far-UV CD spectra of *Ye*SodB revealed high content of α-helix (44%) that was in agreement to the 50% α-helix found in Fe-SOD of *Pseudomonas ovalis* [47]. Changes in the optimum pH of the respective SODs resulted in significant loss of ordered secondary structure, which manifested as corresponding decrease in the enzyme activity.

NADPH-mediated reduction of paraquat generates an intracellular flux of O_2_^−^ that induces toxicity [64]. Farr *et al.* [18] have previously reported that *E. coli* SOD double mutant (strain PN134) showed high sensitivity to paraquat that was reversed by introducing a copy of functional *sod* gene. *E. coli* PN134 strains expressing *Ye*SodA or *Ye*SodB also showed increased resistance to paraquat when compared to the parental strain and these results were similar to those observed by Leclere *et al.* [12]. Maize Mn-SOD expressed in SOD-deficient *S. cerevisiae* has been shown to protect the cells against oxidative stress whereas its over-expression in *Caenorhabditis elegans* and *Drosophila melanogaster* augmented their life span [65], [66], [67]. In the same vein, the *Ye*SODs almost normalized the growth of *E. coli* mutant strain under conditions of oxidative stress by scavenging the exogenous ROS.

### Conclusions

This work represents the first report on the distribution and detailed characterization of superoxide dismutases from *Y*. *enterocolitica* biovar 1A. Most strains expressed both SodA and SodB, whereas, SodC was not expressed by any of the strains used in this study. The recombinant *Ye*SodA and *Ye*SodB were expressed as tetramer and monomer respectively. Complementation of an *E. coli* SOD double mutant with *Ye*SodA and *Ye*SodB enabled *E. coli* to grow under oxidative stress. This coupled with the stability of the *Ye*SODs at low pH and temperature suggested their possible role in conditions such as acidic pH and oxidative stress that are encountered by the organism inside the phagolysosome and refrigerated food respectively.

## Supporting Information

Figure S1 **Phylogenetic (Neighbour Joining) tree:** Constructed using *Ye*SODs amino acid sequences (**a.** SodA, **b.** SodB and **c.** SodC) and other bacterial SOD sequences retrieved from NCBI database. The digit at each branch point represents percentage bootstrap support calculated from 1000 replicates. The trees were constructed by the neighbor joining method in MEGA 4.0 package.
(EPS)

File S1 **Includes Table S1 and S2.** Table S1. Details of *Y.* *enterocolitica*, *Y. intermedia* and *Y. frederiksenii* strains used in the study. Table S2. Distribution of *sodA, sodB* and *sodC* genes and their expression amongst strains of *Yersinia* spp. used in this study.
(DOCX)
