# Supplementary material for: Detection, Distribution and Characterization of Novel Superoxide Dismutases from Yersinia enterocolitica Biovar 1A
Source: PLoS One. 2013 May 21;8(5):e63919. doi: 10.1371/journal.pone.0063919 (PMC3660340; doi:10.1371/journal.pone.0063919)
Supplement: File S1 — Includes Table S1 and S2. Table S1. Details of Y. enterocolitica, Y. intermedia and Y. frederiksenii strains used in the study. Table S2. Distribution of sodA, sodB and sodC genes and their expression amongst strains of Yersinia spp. used in this study. (DOCX) [file pone.0063919.s002.docx]

**Table S1. Details of *Y. enterocolitica, Y. intermedia* and *Y. frederiksenii* strains used in the study**

| **S. No.** | **Strain** | **Biotype** | **Serotype** | **Source** |
| --- | --- | --- | --- | --- |
| ***Y. enterocolitica*** | | | | |
|  | IP27359 | 1A | O:6,30-6,31 | Human |
|  | IP27360 | 1A | O:6,30-6,31 | Human |
|  | IP27362 | 1A | O:6,30-6,31 | Human |
|  | IP27363 | 1A | O:6,30-6,31 | Human |
|  | IP27364 | 1A | O:6,30-6,31 | Human |
|  | IP27366 | 1A | O:6,30-6,31 | Human |
|  | IP26310 | 1A | O:6,30-6,31 | Human |
|  | IP26311 | 1A | O:6,30-6,31 | Wastewater |
|  | IP26312 | 1A | O:6,30-6,31 | Wastewater |
|  | IP26315 | 1A | O:6,30-6,31 | Wastewater |
|  | IP26317 | 1A | O:6,30-6,31 | Wastewater |
|  | IP27403 | 1A | O:6,30 | Wastewater |
|  | IP27405 | 1A | O:6,30 | Human |
|  | IP27407 | 1A | O:6,30 | Human |
|  | IP27425 | 1A | O:6,30 | Human |
|  | IP27427 | 1A | O:6,30 | Human |
|  | IP27429 | 1A | O:6,30 | Human |
|  | IP27431 | 1A | O:6,30 | Human |
|  | IP27433 | 1A | O:6,30 | Human |
|  | IP27434 | 1A | O:6,30 | Human |
|  | IP26260 | 1A | O:6,31 | Human |
|  | IP26305 | 1A | O:10-34 | Wastewater |
|  | IP26314 | 1A | O:10-34 | Wastewater |
|  | E1281580 | 1A | O:15 | Wastewater |
|  | IP26316 | 1A | O:41,42 | Wastewater |
|  | E1281550 | 1A | O:41,43 | Wastewater |
|  | IP26152 | 1A | O:7,8-8-8,19 | Wastewater |
|  | IP26153 | 1A | O:7,8-8-8,19 | Pork |
|  | - | 1A | ND | Pork |
|  | - | 1A | ND | Pig throat |
|  | - | 1A | ND | Pig throat |
|  | - | 1A | ND | Pig throat |
|  | IP27361 | 1A | O:6,30-6,31 | Pig throat |
|  | IP27404 | 1A | O:6,30 | Human |
|  | IP27406 | 1A | O:6,30 | Human |
|  | IP27408 | 1A | O:6,30 | Human |
|  | IP27426 | 1A | O:6,30 | Human |
|  | IP27430 | 1A | O:6,30 | Human |
|  | IP27432 | 1A | O:6,30 | Human |
|  | IP27484 | 1A | O:6,30 | Human |
|  | IP26144 | 1A | O:6,30-6,31 | Human |
|  | IP26147 | 1A | O:10-34 | Wastewater |
|  | - | 1A*^a^* | O:6,30 | Wastewater |
|  | - | 1A *^a^* | O:6,30 | Human |
|  | IP27938 | 1A | O:6,30 | Human |
|  | IP27879 | 1A | O:6,30 | Human |
|  | IP27648*^b^* | 1A | O:10-34 | Human |
|  | IP27387 | 1A | NAG | Human |
|  | IP27388 | 1A | NAG | Human |
|  | IP27485 | 1A | NAG | Human |
|  | 8081*^c^* | 1B | O:8 | Human |
|  | W22703*^d^* | 2 | O:9 | Human |
|  | IP26329 *^b:^* | 2 | O:9 | Human |
|  | IP134 *^b^* | 4 | O:3 | Human |
| ***Y. intermedia*** | | | | |
|  | IP27477 | 2 | NAG |  |
|  | IP72478 | 1 | O:7,8-8 |  |
|  | IP27479 | 1 | NAG |  |
| ***Y.***  ***frederiksenii*** | | | | |
|  | IP27388 | - | NAG |  |
|  | IP27389 | - | O:35 |  |

IP: Institut Pasteur; E: Central Public Health Laboratory (CPHL), Colindale, London; NAG: Non-agglutinable; ND: Not determined.

*^a^:* Strains procured from J. Heesemann *(*Max Von Pettenkofer Institut, Munich, Germany*)*

*^b^*: Strains provided by Elisabeth Carniel (*Yersinia* National Reference Laboratory and WHO Collaborating Center, Pasteur Institute, Paris, France)

*^c^* Strain gifted by M. Skurnik (Department of Bacteriology and Immunology, Haartman Institute, University of Helsinki and Helsinki University Central Hospital Laboratory Diagnostics, Helsinki, Finland)

*^d^* Strain provided by G. Cornelis (Biozentrum der Universitat Basel, Klingelbergstrasse, Switzerland)

**Table S2. Distribution of *sodA, sodB* and *sodC* genes and their expression amongst strains of *Yersinia* spp. used in this study**

| **S. No.** | **Strain Number** | **Detection by PCR** | | | **Zymogram analysis** |
| --- | --- | --- | --- | --- | --- |
|  | | ***sodA*** | ***sodB*** | ***sodC*** |  |
|  | IP27359 | + | + | + | SodA and SodB |
|  | IP27360 | + | + | + | SodA and SodB |
|  | IP27362 | + | + | + | SodA and SodB |
|  | IP27363 | + | + | + | SodA and SodB |
|  | IP27364 | + | + | + | SodA and SodB |
|  | IP27366 | + | + | + | SodA and SodB |
|  | IP26310 | + | + | + | SodA and SodB |
|  | IP26311 | + | + | + | SodA and SodB |
|  | IP26312 | + | + | + | SodA and SodB |
|  | IP26315 | + | + | + | SodA and SodB |
|  | IP26317 | + | + | + | SodA and SodB |
|  | IP27403 | + | + | + | SodA and SodB |
|  | IP27405 | + | + | + | SodA and SodB |
|  | IP27407 | + | + | + | SodA and SodB |
|  | IP27425 | + | + | + | SodA and SodB |
|  | IP27427 | + | + | + | SodA and SodB |
|  | IP27429 | + | + | + | SodA and SodB |
|  | IP27431 | + | + | + | SodA and SodB |
|  | IP27433 | + | + | + | SodA |
|  | IP27434 | + | + | + | SodA and SodB |
|  | IP26260 | + | + | + | SodA and SodB |
|  | IP26305 | + | + | + | SodA and SodB |
|  | IP26314 | + | + | + | SodA |
|  | E1281580 | + | + | + | SodA and SodB |
|  | IP26316 | + | + | + | SodA and SodB |
|  | E1281550 | + | + | + | SodA and SodB |
|  | IP26152 | + | + | + | SodA and SodB |
|  | IP26153 | + | + | + | SodA and SodB |
|  | - | + | + | + | SodA |
|  | - | + | + | + | SodA and SodB |
|  | - | + | + | + | SodA and SodB |
|  | - | + | + | + | SodA and SodB |
|  | IP27361 | + | + | + | SodA and SodB |
|  | IP27404 | + | + | + | SodA |
|  | IP27406 | + | + | + | SodA and SodB |
|  | IP27408 | + | + | + | SodA and SodB |
|  | IP27426 | + | + | + | SodA and SodB |
|  | IP27430 | + | - | + | SodA |
|  | IP27432 | + | + | + | SodA and SodB |
|  | IP27484 | + | + | + | SodA and SodB |
|  | IP26144 | + | + | + | SodA and SodB |
|  | IP26147 | + | + | + | SodA and SodB |
|  | - | + | + | + | SodA |
|  | - | + | + | + | SodA and SodB |
|  | IP27938 | + | + | + | SodA |
|  | IP27879 | + | + | + | SodA and SodB |
|  | IP27648 | + | + | + | SodA and SodB |
|  | IP27387 | + | + | + | SodA and SodB |
|  | IP27388 | + | + | + | SodA and SodB |
|  | IP27485 | + | + | + | SodA and SodB |
|  | W22703 | + | + | + | SodA |
|  | IP26329 | + | + | + | SodA and SodB |
|  | IP134 | + | + | + | SodA |
|  | 8081 | + | + | + | SodA and SodB |
|  | IP27477 | + | + | + | SodA and SodB |
|  | IP72478 | + | + | + | SodA and SodB |
|  | IP27479 | + | + | + | SodA and SodB |
|  | IP27388 | + | + | - | SodA and SodB |
|  | IP27389 | + | + | - | SodA and SodB |

‘+’: Presence of the PCR amplicon. ‘–‘: no PCR amplification observed
